# Ultrasound gap measurement after acute Achilles rupture is reliable overall but uncertain near a 5—mm decision threshold

**DOI:** 10.1007/s00256-026-05243-x

**Published:** 2026-05-08

**Authors:** Dan Mocanu, Katarzyna Bokwa-Dąbrowska, Elin Larsson, Katarina Nilsson Helander, Pawel Szaro

**Affiliations:** 1 Department of Radiology, Institute of Clinical Sciences, Sahlgrenska Academy, University of Gothenburg, Göteborgsvägen 31, 431 80 Gothenburg, Sweden; 2 Department of Radiology, Sahlgrenska University Hospital, Region Västra Götaland, Göteborgsvägen 31, 431 80 Gothenburg, Sweden; 3 The Department of Orthopedics, Sahlgrenska University Hospital Mölndal, Göteborgsvägen 31, 431 80 Gothenburg, Sweden; 4 Department Orthopedics, Institute of Clinical Sciences, Sahlgrenska Academy, University of Gothenburg, Göteborgsvägen 31, 431 80 Gothenburg, Sweden

**Keywords:** Achilles tendon rupture, Ultrasonography, Tendon gap measurement, Interobserver reliability, Intraclass correlation coefficient, Decision threshold

## Abstract

**Objective:**

Ultrasound gap distance has been proposed as a supportive triage input within protocol-based pathways after acute Achilles tendon rupture. We assessed intra-/inter-rater reliability of gap measurement and the extent of classification uncertainty around a 5—mm threshold.

Retrospective observer-agreement study without a definitive reference standard, using protocol-acquired stored ultrasound cine loops from 30 clinically diagnosed complete acute ruptures in a prospectively collected cohort.

**Methods:**

Retrospective observer-agreement study without a definitive reference standard, using protocol-acquired stored ultrasound cine loops from 30 clinically diagnosed complete acute ruptures in a prospectively collected cohort. Two blinded musculoskeletal radiologists re-reviewed stored cine loops and measured tendon-gap distance in two sessions each (≥ 4 weeks apart). We calculated intraclass correlation coefficients (ICC), Bland–Altman mean difference and 95% limits of agreement (LoA), and standard error of measurement (SEM). Agreement at the 5—mm protocol cut-point was assessed using Cohen’s κ and Gwet’s AC1. Using SEM, we derived 90% confidence zones indicating firm classification versus a grey zone where repeat measurement or second read may be considered.

**Results:**

Intra-rater ICC(2,1) was 0.88 (95% CI 0.83–0.91) and 0.94 (0.82–0.97); SEMs were 1.57 and 1.19 mm. Inter-rater bias was + 1.12 mm with LoA − 3.85 to 6.10 mm. Agreement for study experimental pathway classification at the 5—mm cut-point (operative vs non-operative) was moderate (κ = 0.60; AC1 = 0.61), with 6/30 examinations classified differently. SEM-based 90% zones were ≤ 2.42 and ≥ 7.58 mm (rater 1) and ≤ 3.04 and ≥ 6.96 mm (rater 2); a conservative cross-rater rule suggested ≤ 2.05 or ≥ 7.95 mm.

**Conclusion:**

Gap measurement shows good relative reliability, but uncertainty near 5 mm can alter classification. Reporting LoA/SEM alongside ICC and flagging a grey zone may reduce misclassification in borderline cases.

**Supplementary Information:**

The online version contains supplementary material available at 10.1007/s00256-026-05243-x.

## Introduction

Acute Achilles tendon rupture is primarily a clinical diagnosis, with imaging used as required [1, 2]. Early classification guides operative versus non-operative treatment, and ultrasound may be used as an adjunct to clinical assessment and, in some pathways, to support triage [3, 4]. Ultrasound gap distance has been proposed as one supportive input when applying fixed cut-points; under this approach, measurement uncertainty becomes clinically relevant for borderline values close to the threshold [5–7]. Two cut-points are most frequently reported, 10 mm and 5 mm, and have been used to support protocol-based classification to operative versus non-operative management [5–12]. However, the reproducibility of gap measurement, especially around the commonly used 5—mm cut-point, remains insufficiently validated.

Existing studies on ultrasound in Achilles rupture have focused on diagnostic performance or associations with clinical outcomes, which does not directly address the reliability of gap measurement as a quantitative parameter. Moreover, the limited literature on reliability has typically emphasized relative agreement using intraclass correlation coefficients (ICC). While ICC is useful for summarizing rank-order agreement, it does not indicate how often near-threshold cases may be reclassified when a continuous measurement is dichotomized at a fixed cut-point [13]. For clinical pathways that use a 5—mm threshold, absolute measurement error and decision-level agreement are more informative: Bland–Altman limits of agreement (LoA) quantify expected differences between repeated measurements, and the standard error of measurement (SEM) describes the precision of a single reading. Yet SEM and LoA are rarely reported, and there is little evidence on whether between-rater variability remains acceptably low within the narrow range that separates protocol-based operative from non-operative classification.

Ultrasound assessment early after rupture can be challenging because stump margins may be obscured by edema, hematoma, overlapping structures, and irregular tendon ends. Stored cine loops may be re-reviewed during treatment planning, particularly for borderline measurements. Re-review typically involves selecting the frame showing the largest visible separation and measuring the shortest edge-to-edge distance between stump margins. Even when acquisition follows a standardized protocol, variability introduced during re-review (frame selection and caliper placement) may lead to inter-rater differences that cross clinically relevant thresholds. The primary aim of this study was to quantify intra- and inter-rater reliability of ultrasound gap measurement during blinded re-review of stored cine loops acquired using a standardized study protocol. A secondary aim was to quantify uncertainty in threshold-based classification around a 5—mm cut-point and define a pragmatic grey zone where repeat measurement or second read may be considered.

## Methods

### Ethical considerations

Informed consent was obtained from all individual participants included in the study. The study was approved by the National Ethical Review Authority (2019—05457). All procedures conformed to the ethical standards of the institutional and national research committee and with the 1964 Helsinki Declaration and its later amendments.

### Study design

This was a retrospective, blinded re-reading reliability study embedded within the prospective Diagnostic Ultrasound for the Selection of Treatment of Acute Achilles Tendon Rupture (DUSTAR) cohort. The present analyses quantify variability introduced during re-review of stored cine loops (frame selection and caliper placement) and do not capture variability from new image acquisition. Because no definitive reference standard for the true tendon gap was available, the study evaluates reliability/agreement rather than accuracy. The DUSTAR project investigates the role of ultrasound in treatment decision-making for Achilles tendon injuries and includes patients presenting with acute Achilles tendon rupture to the emergency department at our institution between August 1, 2020, and February 12, 2022. Acute Achilles tendon rupture is primarily a clinical diagnosis, established by an orthopedic surgeon in the emergency department. Within DUSTAR, ultrasound was used as a triage tool to support early management planning (operative vs non-operative), not as the primary diagnostic test for complete Achilles tendon rupture. All measurements in this substudy were obtained from saved ultrasound cine loops acquired in routine clinical care; therefore, re-review reliability estimates reflect variability from image selection and caliper placement during re-review rather than variability from new image acquisition at the scanner. The study adhered to the Guidelines for Reporting Reliability and Agreement Studies (GRRAS)[14].

### Setting and participants

This substudy of the larger DUSTAR study was conducted at a single tertiary academic musculoskeletal imaging center. This reliability substudy included the first 30 consecutive eligible DUSTAR patients with protocol-acquired cine loops. Patients were eligible for inclusion if they were between 16 and 65 years of age, presented with a closed mid-substance Achilles tendon rupture, and underwent initial treatment within 48 h of injury. Exclusion criteria included a history of prior Achilles tendon rupture (regardless of side), concomitant injuries affecting foot or lower leg function, diabetes mellitus, neurovascular disease, immunosuppressive therapy, or inability to understand the Swedish language.

All eligible patients underwent standardized ultrasound assessment within 48 h of inclusion while attending the emergency department. During the diagnostic process, patients were temporarily immobilized in a below-knee plaster cast with the foot placed in approximately 30 of plantar flexion. The ultrasound examination was performed in the radiology department at our center.

### Ultrasound acquisition and image storage

After clinical diagnosis of a complete rupture, initial treatment began with immobilization in a ventral below-knee cast. Ultrasound was performed within 48 h with the cast in place, consistent with the clinical workflow. Patients were examined prone with the knee flexed approximately 10, and the foot supported by a wedge cushion to achieve approximately 30° plantarflexion. Thus, all gap measurements in this study reflect the tendon position in plantarflexion. All examinations were performed in routine care by the on-duty radiologist using a written, standardized DUSTAR acquisition protocol. Before study start, on-duty radiologists were trained in the protocol and instructed to acquire long-axis (sagittal) cine loops with the probe aligned to the tendon fibers and centered over the rupture gap, ensuring visualization of both proximal and distal stump margins within the same field of view. Before each examination, the on-duty radiologist reviewed the written DUSTAR acquisition protocol. Ultrasound was performed using a LOGIQ E9 system (GE Healthcare) with a 6–15 MHz linear probe. The Achilles tendon was assessed from the musculotendinous junction to the calcaneal insertion in both long- and short-axis planes, and 2–4 standardized cine loops spanning the rupture region were stored for subsequent re-review. Dynamic maneuvers were not performed because immobilization limited ankle motion. This substudy evaluates variability introduced during image re-review (frame selection and caliper placement) on stored cine loops and does not quantify variability from new image acquisition.

### Tendon-gap measurement and re-review procedure

Stored ultrasound cine loops were independently re-reviewed by two musculoskeletal radiologists. For each examination, the reader selected the frame showing the largest visible separation between proximal and distal tendon stump margins on the long-axis view and measured the gap as the shortest edge-to-edge distance between stump margins using electronic calipers (mm). Stump margins were defined as the visible echogenic tendon ends on long-axis imaging; when ends were opposed or overlapping on the selected frame, the gap was recorded as 0 mm. Measurements therefore reflect imaging obtained with the ankle immobilized in approximately 30° plantarflexion with the ventral cast in place; measurements were not performed in neutral or dorsiflexion. Each rater performed two re-review sessions separated by ≥ 4 weeks and was blinded to the other rater’s measurements and to their own prior measurements. No new ultrasound scanning was performed for the purposes of this substudy. In the DUSTAR pathway, a 5—mm cut-point was applied for protocol-based classification into operative versus non-operative management; this cut-point was used in the present analyses to assess threshold agreement.

### Raters

Two musculoskeletal radiologists, each with ten years of experience in ultrasound, independently re-reviewed all examinations. Both raters were blinded to each other’s measurements, the original radiology reports, clinical data, and any previously selected image frames. Case order was independently re-randomized per session; raters were blinded to their own prior measurements. Each rater performed two separate gap measurements for every patient, with a washout interval of at least four weeks between sessions. For each measurement, raters selected the frame they considered showing the largest visible separation and used electronic calipers to measure the shortest distance between tendon ends in millimeters.

### Outcomes

The primary outcome was the tendon gap (mm). We quantified intra- and inter-rater reliability and agreement.

Secondary outcomes were (1) decision-level agreement for DUSTAR protocol-based classification (operative vs non-operative) using the 5—mm operational cut-point; (2) “grey-zone” frequencies where single-read decisions are most error-prone; (3) systematic bias between raters and sessions.

Decision-level agreement at 5 mm was summarized with Cohen’s κ and Gwet’s AC1. We report the prevalence of ≥ 5—mm classifications and provide both statistics with 95% CIs.

To relate measurement error to threshold classification, we used the SEM to define an uncertainty range around 5 mm where a single measurement is most likely to change category on repeat reading. We verified the grey-zone conclusions using log-scale (ratio) analyses and by restricting analyses to measurements between 0 and 10 mm; conclusions were unchanged.

We used 5 mm as the decision threshold because it is the pre-specified operational cut-point in the DUSTAR pathway.

### Statistical analysis

Re-review reliability was assessed with the intraclass correlation coefficient (ICC) using a two-way random-effects, absolute-agreement, single-measure model [ICC(2,1)]. Inter-rater reliability and agreement were calculated using, for each participant, the mean of each rater’s two sessions (unit of analysis: participant). Intra-rater metrics were based on each rater’s session-specific measurements. Sensitivity analyses repeating inter-rater metrics using (1) session-matched pairs and (2) all reads in a mixed-effects model produced materially similar results. ICCs were interpreted using Koo & Li (2016): poor < 0.50, moderate 0.50–0.74, good 0.75–0.89, and excellent ≥ 0.90[15].

Sample size justification (precision-based). This embedded reliability study was designed to estimate reliability with acceptable precision rather than to test a hypothesis. With n = 30 and an expected ICC of ~ 0.85, the 95% confidence interval (CI) width is approximately 0.20, which we considered sufficient for feasibility and for interpreting decision thresholds. For agreement at the 5—mm cut-point, *n* = 30 yields wider CIs for κ/AC1; therefore, we report CIs together with absolute counts.

Absolute agreement was evaluated using Bland–Altman analysis. We report the bias (mean difference) and 95% limits of agreement (LoA = bias ± 1.96 × SD of the differences). The standard error of measurement (SEM), representing the expected absolute error of a single observation, was estimated from the two sessions per rater as[16]:

$$SEM=SD\left(A-B\right)/\sqrt{2},$$

where A and B are repeated readings in the same participant. The minimal detectable change at 95% confidence was calculated as MDC_95 = 1.96 × √2 × SEM[17].

To relate measurement precision to threshold-based decisions, we defined confidence bounds around the 5—mm cut-point as 5 ± z·SEM (*z* = 1.28, 1.64, and 1.96 for 80%, 90%, and 95% certainty, respectively). Values between these bounds were considered a grey zone where repeat measurement or consensus review may be appropriate. We also estimated the probability that the true gap is ≥ 5 mm given an observed value x under a normal error model:

$$Pr\left(\left.true\ge 5\right|x\right)=1-\Phi \left(\left(5-x\right)/SEM\right),$$

where Φ is the standard normal cumulative distribution function.

All analyses were performed in R version 4.4.3 (R Foundation for Statistical Computing, Vienna, Austria). The irr, psych, and blandr packages were used for ICC and Bland–Altman analyses. Figures were created in R using ggplot2 based on the study dataset. No third-party photos, icons, templates, stock images, or online graphic elements were used. The figures have not been published previously.

A large language model (ChatGPT) was used only for grammar, syntax, and language editing. All authors reviewed and verified the final manuscript and take full responsibility for its content.

## Results

### Study cohort

**Fig. 1:**
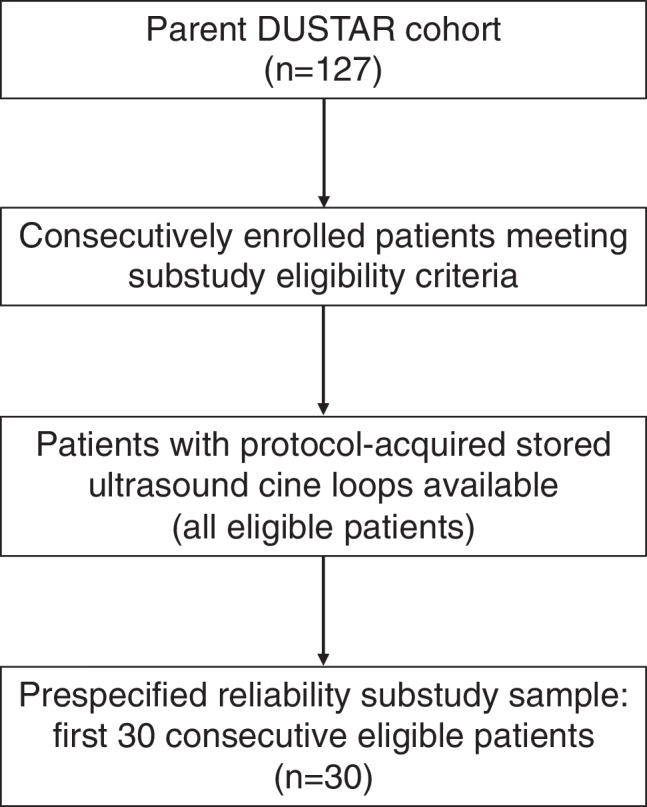
Participant flow diagram for the reliability substudy. Patients were consecutively enrolled in the parent DUSTAR cohort (*n* = 127). The reliability substudy was prespecified to include the first 30 consecutive eligible patients with protocol-acquired cine loops; remaining cohort patients were outside the scope of this substudy

A total of 30 patients were included in the study. Of these, 24 were male (80%), and the mean (standard deviation, SD) age of the cohort was 45 (10.5) years (Table 1). Participant flow from the parent DUSTAR cohort to the reliability substudy sample is shown in Fig. 1.

**Table 1:** Demographics of the patients included

	All (*n* = 30)
Age, mean (SD)	45 (10.5)
Sex, male (*n* (%))	24 (80%)
female (*n* (%))	6 (20%)
Treatment (percentage operative treatment)	63%
Side (percentage right)	53%

*SD* standard deviation

### Intra-rater re-review reliability

#### Rater 1

According to measurements done by Rater 1 the tendon gap measurements ranged from 0.0 mm to 18 mm (Supplementary material Fig. S1). Values of 0 mm reflect complete ruptures with tendon ends apposed or overlapping tendon ends (no measurable separation on the selected frame).

The intra-rater re-review reliability was good with an intraclass correlation coefficient ICC(2,1) = 0.88 (95% CI: 0.83–0.91).

**Fig. 2:**
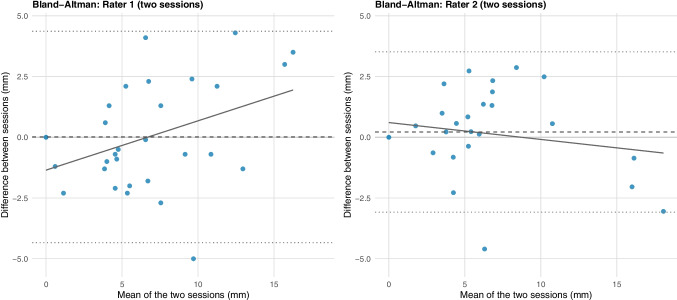
Bland–Altman plots for intra-rater repeatability with matched axes. Left: Rater 1 (two sessions) bias 0.01 mm [95% Confidence Interval (CI) −0.78 to 0.81]; 95% limits of agreement (LoA) −4.34 to 4.37 mm (limits' 95% CIs: −5.71 to −2.97; 2.99 to 5.74). Right: Rater 2 (two sessions)—bias 0.22 mm [95% CI −0.39 to 0.82]; 95% LoA −3.08 to 3.52 mm (limits' 95% CIs: −4.13 to −2.04; 2.48 to 4.56). Dashed line = bias; dotted lines = 95% LoA; linear fit shown to assess proportional bias

The mean difference between repeated measurements was 0.01 mm, with 95% LoA from −4.34 to 4.37 mm (Fig. 2). The SEM was 1.57 mm. The MDC95 was 4.34 mm, indicating that changes smaller than ~ 4.3 mm are within expected measurement noise for a single rater.

**Fig. 3:**
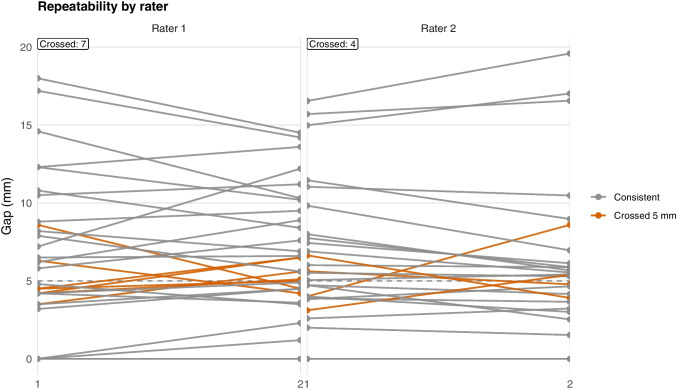
Paired measurements (mm) performed by Rater 1 and Rater 2 during two reading sessions

**Fig. 4:**
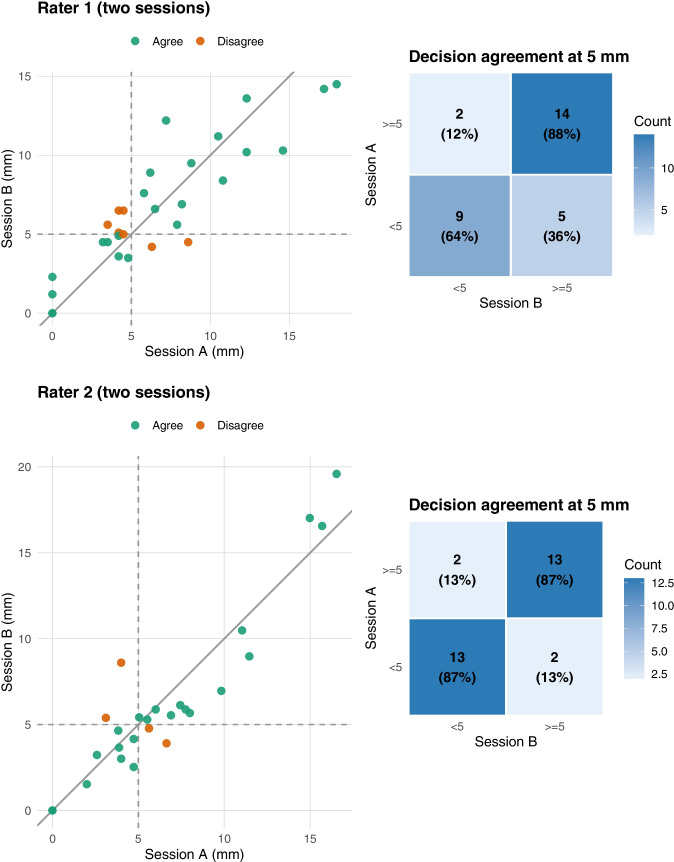
Intra-rater decision panels. Each rater shows: Left: first session vs second session identity scatter with 5—mm dashed cut lines; green points = consistent threshold-based classification (< 5 or ≥ 5 mm), orange points = within-rater disagreement. Right: 2 × 2 heatmap (rows = Session A—the first session, columns = Session B—the second session) with counts and row-percentages

In seven cases threshold-based classification (< 5 vs ≥ 5 mm) differed between the two reading sessions (Figs. 3 and 4; Supplementary Fig. S2). Intra-rater decision agreement was moderate (Cohen’s κ = 0.525, 95% CI 0.200—0.800; Gwet’s AC1 = 0.546, 95% CI 0.214—0.824; Fig. 4).

#### Rater 2

Tendon gap measurements ranged from 0.0 mm to 19.6 mm (Supplementary material Fig. S1). Rater 2 demonstrated higher intra-rater re-review reliability than Rater 1 and was excellent, ICC(2,1) = 0.94 (95% CI: 0.82–0.97).

The mean difference between repeated measurements was 0.22 mm, with 95% LoA from −3.08 to 3.52 mm (Fig. 2). The SEM was 1.19 mm. The MDC95 was 3.30 mm, indicating that changes smaller than ~ 3.3 mm may reflect measurement noise.

In 4 cases, threshold-based classification (< 5 vs ≥ 5 mm) differed between the two reading sessions (Fig. 3 and 4; Supplementary Fig. S2).

Intra-rater decision agreement was substantial (Cohen's kappa = 0.733 (95% CI 0.461–0.933); Gwet’s AC1 = 0.733 (95% CI 0.476–0.937)), Fig. 4.

### Directional bias

**Fig. 5:**
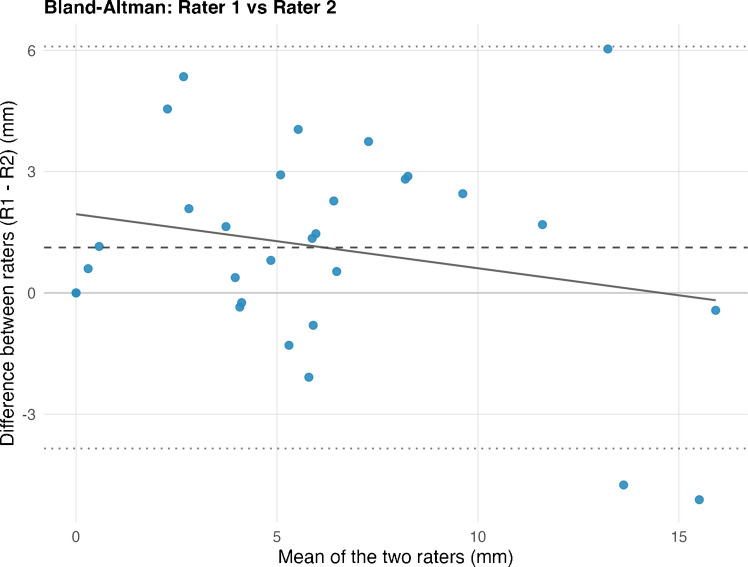
Bland–Altman plot for inter-rater agreement. Points compare the mean of the two raters to their difference (R1—R2). Dashed line = bias; dotted lines = 95% limits of agreement. Bias 1.12 mm [95% Confidence Interval (CI) 0.22 to 2.03]; 95% Limits of agreement (LoA) −3.85 to 6.10 mm (limits' 95% CIs: −5.42 to −2.28; 4.53 to 7.66)

Rater 1 measured larger gaps than Rater 2 in two-thirds of cases (20/30; 66.7%), with a mean bias of + 1.12 mm (R1–R2), while R1 measured smaller in 8/30 (26.7%) and ties occurred in 2/30 (6.7%) (Figs. 3 and 5; Supplementary Fig. S2).

Within raters, patterns were asymmetric: Rater 1’s first session read lower than the second in 17/30 cases (56.7%) and higher in 11/30 (36.7%; ties 2/30), whereas Rater 2’s first session read higher than the second in 16/30 (53.3%) and lower in 8/30 (26.7%; ties 6/30) (Fig. 3, Supplementary Fig. S2).

### Inter-rater re-review reliability

#### Measurements

Inter-rater agreement for measurements of the gap was good ICC(2,1) = 0.82 (95% CI 0.63–0.91)[15].

The mean difference between raters was 1.12 mm, with Bland–Altman LoA ranging from −3.85 to 6.10 mm (Fig. 5). SEM for inter-rater comparison was 1.79 mm. The inter-rater MDC95 was 4.95 mm, i.e., a between-rater difference of ~ 5 mm is required to exceed measurement noise with 95% confidence.

**Fig. 6:**
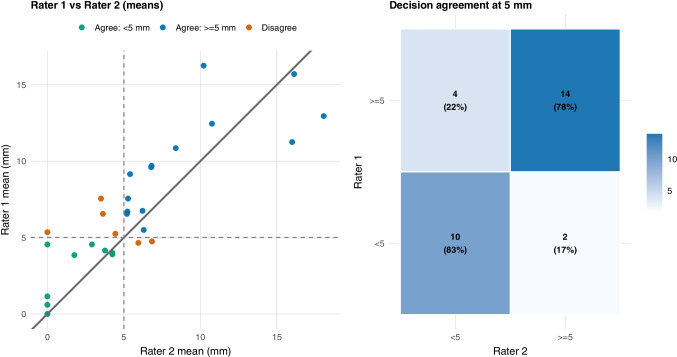
Decision panel. Left: identity scatter of per-patient means (Rater 1 vs Rater 2) with identity line and dashed 5 mm guides; points colored by decision agreement (green = both < 5 mm, blue = both >  = 5 mm, red = disagreement). Right: 2 × 2 confusion heatmap (Rater 1 rows, Rater 2 columns) with counts and row percentages

Six patients had discordant threshold-based classification (< 5 vs ≥ 5 mm) based on the mean tendon gap measurements of Rater 1 and Rater 2 (Fig. 6).

#### Inter-rater agreement in threshold-based classifications

Of 30 cases, 14/30 (46.7%) had a mean gap ≥ 5 mm by both raters; inter-rater decision agreement at the 5—mm threshold was moderate (Cohen’s kappa = 0.595 (95% CI 0.267–0.864)) or substantial (Gwet’s AC1 = 0.607 (95% CI 0.333–0.872)), with 6/30 (20.0%) discordant classifications (Fig. 6).

**Fig. 7:**
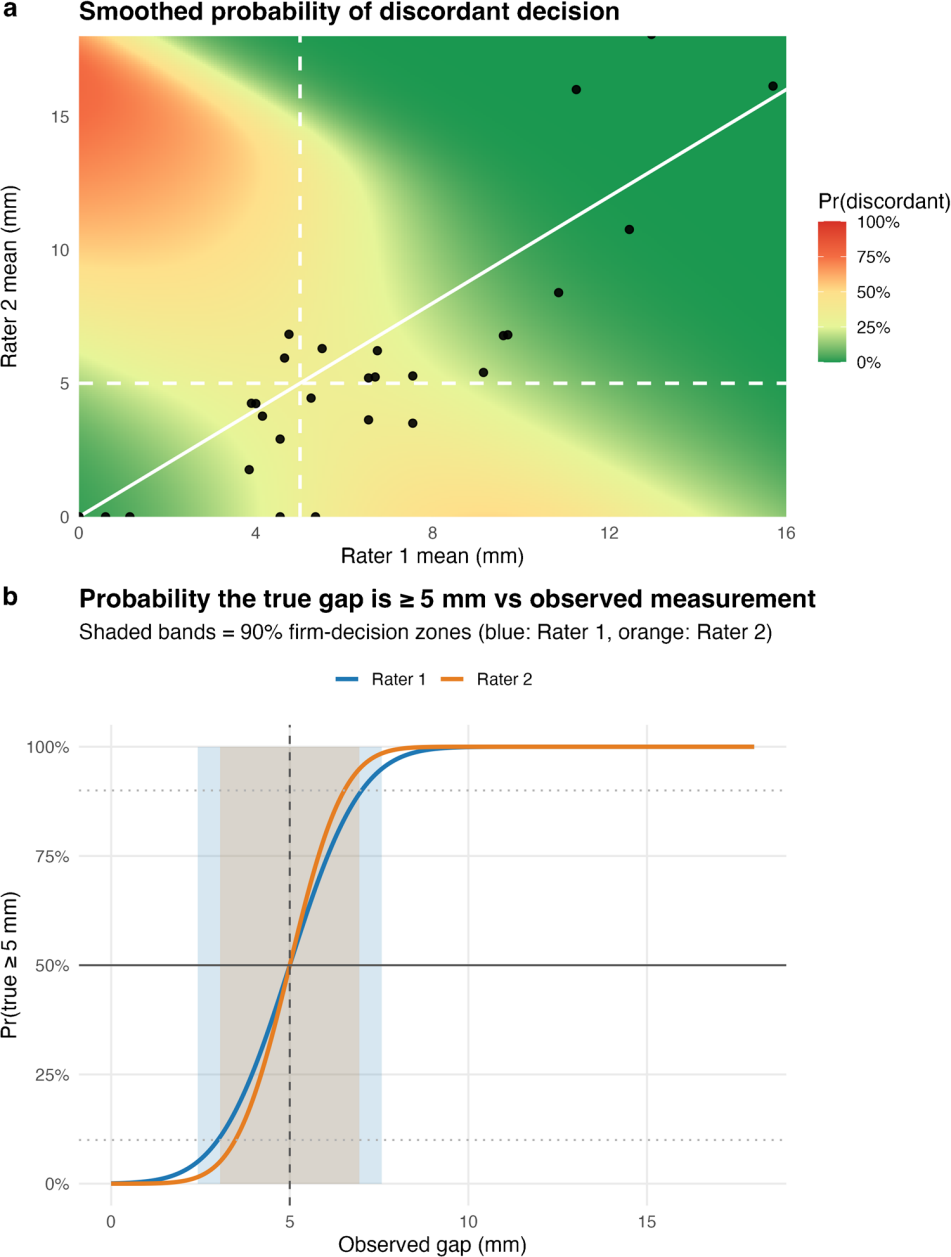
Inter-rater differences are shown two ways: (a) where discordant calls occur on the R1–R2 plane, and (b) how each rater’s measurement maps to the probability the true gap is ≥ 5 mm. **A**) Smoothed map of disagreement across the Rater-1 × Rater-2 measurement plane. Color shows the Gaussian-kernel estimate of Pr(discordant | R1, R2), from 0 (green) to 1 (dark red). Black dots are observed pairs (*n* = 30). Solid line: identity (R1 = R2). Dashed lines: 5—mm cut-point. **B**) Probability that the true gap is ≥ 5 mm versus the observed measurement. Curves show, for each rater, the estimated chance the true gap meets/exceeds 5 mm (normal measurement error; SEM from paired sessions). Shaded regions indicate each rater’s 90% “firm-decision” zone; values between them form a grey zone where repeat/consensus reading is advised. Vertical dashed line: 5—mm threshold. Horizontal dotted lines: 10% and 90% reference levels. Blue: Rater 1; Orange: Rater 2

**Fig. 8:**
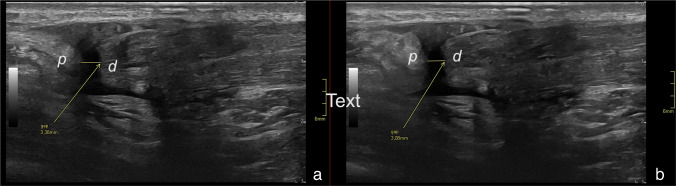
Ultrasound long-axis image demonstrating concordant tendon gap measurement between two raters. Both raters identified similar tendon stump margins and placed calipers in comparable positions, resulting in similar gap measurements (a-Rater 1: 3.4 mm; b- Rater 2: 3.1 mm). The measurements fall on the same side of the 5—mm threshold, leading to consistent classification within the treatment pathway. p = proximal stump; d = distal stump

**Fig. 9:**
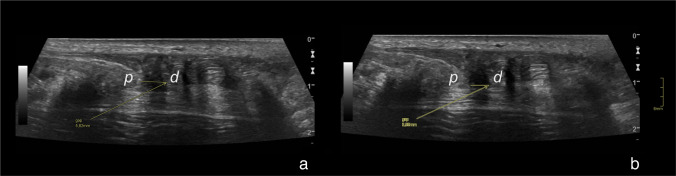
Ultrasound long-axis image demonstrating discordant tendon gap measurement between two raters. Differences in frame selection and/or caliper placement across irregular tendon stump margins resulted in different gap measurements (a—Rater 1: 5.8 mm; b—Rater 2: 4.8 mm). These measurements fall on opposite sides of the 5—mm threshold and would therefore classify the patient into different treatment groups within a protocol-based pathway. *p* = proximal stump; d = distal stump

Discordant classifications clustered around the 5—mm cut-point (Fig. 7a). Inter-rater scatter was symmetric and Bland–Altman regression showed no proportional bias; a fixed bias remained (Rater 1 ≈ + 1.1 mm; Fig. 5). Representative ultrasound examples of concordant and discordant gap measurements with annotated caliper placement are shown in Figs. 8 and 9.

### Proportional bias

Bland–Altman regression showed no proportional bias (slope − 0.134, *p* = 0.225; Fig. 5). Absolute differences increased with magnitude (Spearman ρ = 0.428), so we also report log-scale LoA: ratio bias + 19.4% with ratio LoA − 37.9% to + 129.6%.

### Decision certainty and grey zones

Within-rater SEM was 1.57 mm for Rater 1 and 1.19 mm for Rater 2. Using a 90% criterion (z ≈ 1.64), the resulting confidence cut-offs were: Rater 1: ≤ 2.42 mm (firm non-operative) and ≥ 7.58 mm (firm operative); grey zone 2.42–7.58 mm (Fig. 7b). Rater 2: ≤ 3.04 mm and ≥ 6.96 mm; grey zone 3.04–6.96 mm. A conservative cross-rater rule using the between rater-single measure SEM (1.79 mm) yields ≤ 2.05 mm and ≥ 7.95 mm (grey zone 2.05–7.95 mm). The probability plot shows how certainty increases smoothly with distance from 5 mm and illustrates the wider grey zone for the rater with larger SEM (Fig. 7b).

## Discussion

The most important finding of this study was that ultrasound gap measurement during re-review of stored cine loops showed good relative reliability (ICC), yet only moderate agreement when dichotomized at a 5—mm threshold. This apparent mismatch arises because expected measurement variability (SEM/LoA) is sufficient to shift borderline cases across a fixed cut-point, even when rank-order agreement is high. Discordant classifications clustered around the threshold, and a small fixed inter-rater bias further contributed to reclassification in near-threshold cases.

The precision analyses explained this observation, the within-rater SEMs (1.57 mm and 1.19 mm) and Bland–Altman limits (ca ± 4 mm) indicate that 1—3 mm differences are entirely probable on repeat. Mapping discordance probability over the rater-mean plane confirmed that disagreement is located around 5 mm, with symmetric patterns indicating that discordance reflects threshold proximity rather than systematic over- or under-calling. Nevertheless, we detected a modest between-rater bias (+ 1.12 mm; Rater 1 higher), which can further nudge near-threshold cases across the decision edge. Our results suggest that, when a fixed cut-point is used, near-threshold values may be better interpreted probabilistically rather than as a strict binary rule. Using the observed SEMs, we defined practical “firm decision zones”. For Rater 1, measurements ≤ 2.42 mm or ≥ 7.58 mm imply ≥ 90% certainty for non-operative or operative classification; for Rater 2, ≤ 3.04 mm or ≥ 6.96 mm. A conservative cross-rater approach suggests ≤ 2.05 mm (non-operative) and ≥ 7.95 mm (operative) as ranges where decisions are unlikely to change with repeat reading. Values within the “grey zone” should be treated with caution and double checked, or consensus reviewed.

The rupture gap can be assessed with ultrasound and may influence treatment decisions and clinical outcomes [3, 4, 8, 12]. However, achieving the measurement precision needed, especially around a strict 5—mm threshold can be difficult in routine practice [5]. Evidence that larger gaps predict worse function remains inconsistent across studies. Our findings indicate that part of this inconsistency likely reflects measurement reliability and the decision rules derived from those measurements. If a threshold-based pathway incorporates ultrasound gap measurement, the cut-point should be selected with attention to measurement error and validated prospectively [7, 8, 11, 12].

When a fixed cut-point is used, our results suggest that borderline measurements (approximately 3—7 mm in this dataset) are most prone to disagreement. In such cases, repeat measurement or second read may be considered, and standardized acquisition/reading procedures may reduce variability.

While a prior systematic review reports good to excellent intra- and inter-rater re-review reliability for ultrasound measurement of the Achilles tendon gap, most studies have been based primarily on ICC [5, 18–21]. In contrast, our study used additional methods, including ICC, Bland–Altman and SEM, and, as a novel approach, evaluated decision-level agreement at the threshold-based classifications. This method allowed us to detect the interesting paradox that despite relatively high ICC, agreement at this clinical cut-point was lower. This means that, when using ICC alone, it is not possible to detect that small measurement errors can flip operative versus non-operative classification. Bland–Altman analysis revealed absolute between- and within-rater differences that can change classification near the 5—mm cut-off. ICCs review reproducibility at the group level but miss patient-level uncertainty. Therefore, when a fixed cut-point is used, near-threshold values may warrant repeat measurement or second read, depending on the clinical context. Future reliability studies on Achilles tendon gap should incorporate threshold-based agreement metrics alongside ICC to better reflect decision-making in clinical practice.

These results highlight a limitation of thresholding a continuous ultrasound measurement in acute rupture triage [22]. Ultrasound gap estimates should be interpreted with caution near decision thresholds because expected measurement error may change threshold-based classification (operative vs. non-operative). Saving cine loops enables later re-review and may improve interpretability in borderline cases. Measurement discrepancies in tendon gap are sometimes significant, and their causes remain unknown. It can be related to operator experience, however in our study both raters were very experienced in routine ultrasound. Irregular, frayed stump ends can obstruct consistent landmark identification and likely contribute to variability in measurements.

Our study has limitations. The single-center sample size limits precision and generalizability. Although examinations were re-evaluated under a standardized approach, frame selection and caliper placement remain sources of error. Ultrasound variability arises not only from measurement review but also from acquisition factors (probe angle, settings, examination setup) and artefacts; our re-review design does not capture this component [23]. The probability map is smoothed and least certain in sparse regions, and the inter-rater LoA indicate that individual borderline cases can shift category on repeat measurement. Only two raters re-evaluated the gap. In addition, because no definitive reference standard was available, our findings should be interpreted as observer-agreement estimates and not as evidence of measurement accuracy. Because acquisitions were performed in routine care and were not repeated by multiple radiologists, we do not quantify between-operator acquisition variability, which may further widen uncertainty in other settings. Future work should include multi-reader, multi-center validation and prospective testing of a grey-zone workflow, potentially leveraging fixed-rig, 3D, or extended field-of-view imaging to reduce variability [19, 21]. Finally, dynamic ultrasound assessment was not performed because immobilization in a cast is integral to acute management and restricts ankle motion. Therefore, our findings reflect the reliability of static gap measurements and may not capture additional information obtainable with dynamic assessment. In cases with a measured gap of 0 mm, static ultrasound may not reliably distinguish complete rupture with apposed or overlapping tendon ends from subtotal rupture.

Future studies should include multicenter, multi-rater studies with standardized protocols, and should relate continuous measurements to patient-centered outcomes to refine or replace the 5 mm rule. Decision-curve analysis and Bayesian-probabilistic reporting could quantify net clinical benefit and make uncertainty obvious.

Ultrasound gap measurement on stored cine loops shows good intra- and interobserver reliability, but absolute error remains clinically relevant near a 5—mm cut-point. In borderline measurements, interobserver differences can change threshold-based classification. When a cut-point is used to support the treatment decision, a grey zone around the threshold may help interpret borderline measurements; in such cases, repeat measurement or second read can be considered before finalizing protocol-based classification. Where a cut-point is used within a pathway, borderline results may warrant repeat measurement or second read; prospective studies are needed to test whether such verification improves decision consistency and outcomes.

## Supplementary Information

Below is the link to the electronic supplementary material.

Supplementary file1 (PDF 13 KB)

Supplementary file2 (PDF 21 KB)

## Data Availability

The data supporting this study are available from the corresponding author upon reasonable request, in accordance with the Ethics Committee’s requirements and applicable privacy regulations.

## Abbreviations

AC1: Gwet’s first-order agreement coefficient
CI: Confidence interval
DUSTAR: Diagnostic Ultrasound for the Selection of Treatment of Acute Achilles Tendon Rupture (study name)
GRRAS: Guidelines for Reporting Reliability and Agreement Studies
ICC: Intraclass correlation coefficient
LoA: Limits of agreement
MDC95: Minimal detectable change at 95% confidence
R: R statistical computing environment
SD: Standard deviation
SEM: Standard error of measurement
κ: Cohen’s kappa

## References

[1] Chiodo CP, Glazebrook M, Bluman EM, Cohen BE, Femino JE, Giza E, et al. American academy of orthopaedic surgeons clinical practice guideline on treatment of Achilles tendon rupture. J Bone Joint Surg Am. 2010;92(14):2466–8. 20962199

[2] Park SH, Lee HS, Young KW, Seo SG. Treatment of acute Achilles tendon rupture. Clin Orthop Surg. 2020;12(1):1–8. 32117532 10.4055/cios.2020.12.1.1 PMC7031433

[3] Yang X, Meng H, Quan Q, Peng J, Lu S, Wang A. Management of acute Achilles tendon ruptures: a review. Bone Joint Res. 2018;7(10):561–9. 30464836 10.1302/2046-3758.710.BJR-2018-0004.R2 PMC6215245

[4] Pass B, Robinson P, Ha A, Levine B, Yablon CM, Rowbotham E. The Achilles tendon: imaging diagnoses and image-guided interventions-AJR expert panel narrative review. AJR Am J Roentgenol. 2022;219(3):355–68. 35506554 10.2214/AJR.22.27632

[5] Fenech M, Ajjikuttira A, Edwards H. Ultrasound assessment of acute Achilles tendon rupture and measurement of the tendon gap. Australas J Ultrasound Med. 2024;27(2):106–19. 38784700 10.1002/ajum.12384 PMC11109999

[6] Mubark I, Abouelela A, Arya S, Buchanan D, Elgalli M, Parker J, et al. Achilles tendon rupture: can the tendon gap on ultrasound scan predict the outcome of functional rehabilitation program? Cureus. 2020;12(9):e10298. 33047088 10.7759/cureus.10298 PMC7540077

[7] Qureshi A, Gulati A, Adukia V, Shah A, Mangwani J. The influence of the site of rupture and gap distance in acute Achilles tendon rupture treated with functional rehabilitation. Injury. 2023;54(4):1216–21. 36828734 10.1016/j.injury.2023.02.020

[8] Yassin M, Myatt R, Thomas W, Gupta V, Hoque T, Mahadevan D. Does size of tendon gap affect patient-reported outcome following Achilles tendon rupture treated with functional rehabilitation? Bone Joint J. 2020;102-b(11):1535–41. 33135439 10.1302/0301-620X.102B11.BJJ-2020-0908.R1

[9] Hansen MS, Vestermark MT, Hölmich P, Kristensen MT, Barfod KW. Individualized treatment for acute Achilles tendon rupture based on the Copenhagen Achilles rupture treatment algorithm (CARTA): a study protocol for a multicenter randomized controlled trial. Trials. 2020;21(1):399. 32398120 10.1186/s13063-020-04332-z PMC7218535

[10] Amlang MH, Zwipp H, Friedrich A, Peaden A, Bunk A, Rammelt S. Ultrasonographic classification of Achilles tendon ruptures as a rationale for individual treatment selection. ISRN Orthop. 2011;2011:869703. 24977069 10.5402/2011/869703 PMC4063199

[11] Westin O, Nilsson Helander K, GrävareSilbernagel K, Möller M, Kälebo P, Karlsson J. Acute ultrasonography investigation to predict reruptures and outcomes in patients with an Achilles tendon rupture. Orthop J Sports Med. 2016;4(10):2325967116667920. 27781212 10.1177/2325967116667920 PMC5066526

[12] Elbeshbeshy M, Khalafallah M, Fell A, Davies A, Hashem M. Impact of tendon gap on decision-making in acute Achilles tendon rupture: a systematic review. Cureus. 2025;17(9):e91902. 41080291 10.7759/cureus.91902 PMC12510132

[13] Mehta S, Bastero-Caballero RF, Sun Y, Zhu R, Murphy DK, Hardas B, et al. Performance of intraclass correlation coefficient (ICC) as a reliability index under various distributions in scale reliability studies. Stat Med. 2018;37(18):2734–52. 29707825 10.1002/sim.7679 PMC6174967

[14] Kottner J, Audigé L, Brorson S, Donner A, Gajewski BJ, Hróbjartsson A, et al. Guidelines for reporting reliability and agreement studies (GRRAS) were proposed. J Clin Epidemiol. 2011;64(1):96–106. 21130355 10.1016/j.jclinepi.2010.03.002

[15] Koo TK, Li MY. A guideline of selecting and reporting intraclass correlation coefficients for reliability research. J Chiropr Med. 2016;15(2):155–63. 27330520 10.1016/j.jcm.2016.02.012 PMC4913118

[16] Atkinson G, Nevill AM. Statistical methods for assessing measurement error (reliability) in variables relevant to sports medicine. Sports Med. 1998;26(4):217–38. 9820922 10.2165/00007256-199826040-00002

[17] de Vet HC, Terwee CB, Knol DL, Bouter LM. When to use agreement versus reliability measures. J Clin Epidemiol. 2006;59(10):1033–9. 16980142 10.1016/j.jclinepi.2005.10.015

[18] Thoirs KA, Childs J. Are ultrasound measurements of Achilles tendon size reliable? A systematic review of rater reliability. Ultrasound Med Biol. 2018;44(12):2476–91. 30154035 10.1016/j.ultrasmedbio.2018.07.011

[19] Alabau-Dasi R, Dominguez-Maldonado G, Ortega-Avila AB, Gordillo-Fernandez LM, Ortiz-Romero M, Melchor-Rodriguez JM, et al. Validation of fixed ultrasonography for Achilles tendon assessment: a reliability study. Diagnostics (Basel). 2024. 10.3390/diagnostics14192221. 39410625 10.3390/diagnostics14192221 PMC11476014

[20] Habersack A, Zussner T, Thaller S, Tilp M, Svehlik M, Kruse A. Validity and reliability of a novel 3D ultrasound approach to assess static lengths and the lengthening behavior of the gastrocnemius medialis muscle and the Achilles tendon in vivo. Knee Surg Sports Traumatol Arthrosc. 2022;30(12):4203–13. 35906410 10.1007/s00167-022-07076-2 PMC9668947

[21] Silbernagel KG, Shelley K, Powell S, Varrecchia S. Extended field of view ultrasound imaging to evaluate Achilles tendon length and thickness: a reliability and validity study. Muscles Ligaments Tendons J. 2016;6(1):104–10. 27331037 10.11138/mltj/2016.6.1.104 PMC4915448

[22] Adler RS. What is the place of ultrasound in MSK imaging? Skelet Radiol. 2024;53(9):1699–709. 10.1007/s00256-024-04642-2 38492028

[23] Terslev L, Filippucci E, Torp-Pedersen S. B-mode artefacts relevant in rheumatological musculoskeletal ultrasound—impact on image interpretation and their diagnostic value. Skeletal Radiol. 2025;54(11):2373–83. 40234330 10.1007/s00256-025-04928-z

