# Supplementary figures and images for: Ultrasound gap measurement after acute Achilles rupture is reliable overall but uncertain near a 5—mm decision threshold
Source: Skeletal Radiol. 2026 May 8;55(9):2301–13. doi: 10.1007/s00256-026-05243-x (PMC13369784; doi:10.1007/s00256-026-05243-x)

Raw ultrasound measurements by rater (two sessions each)

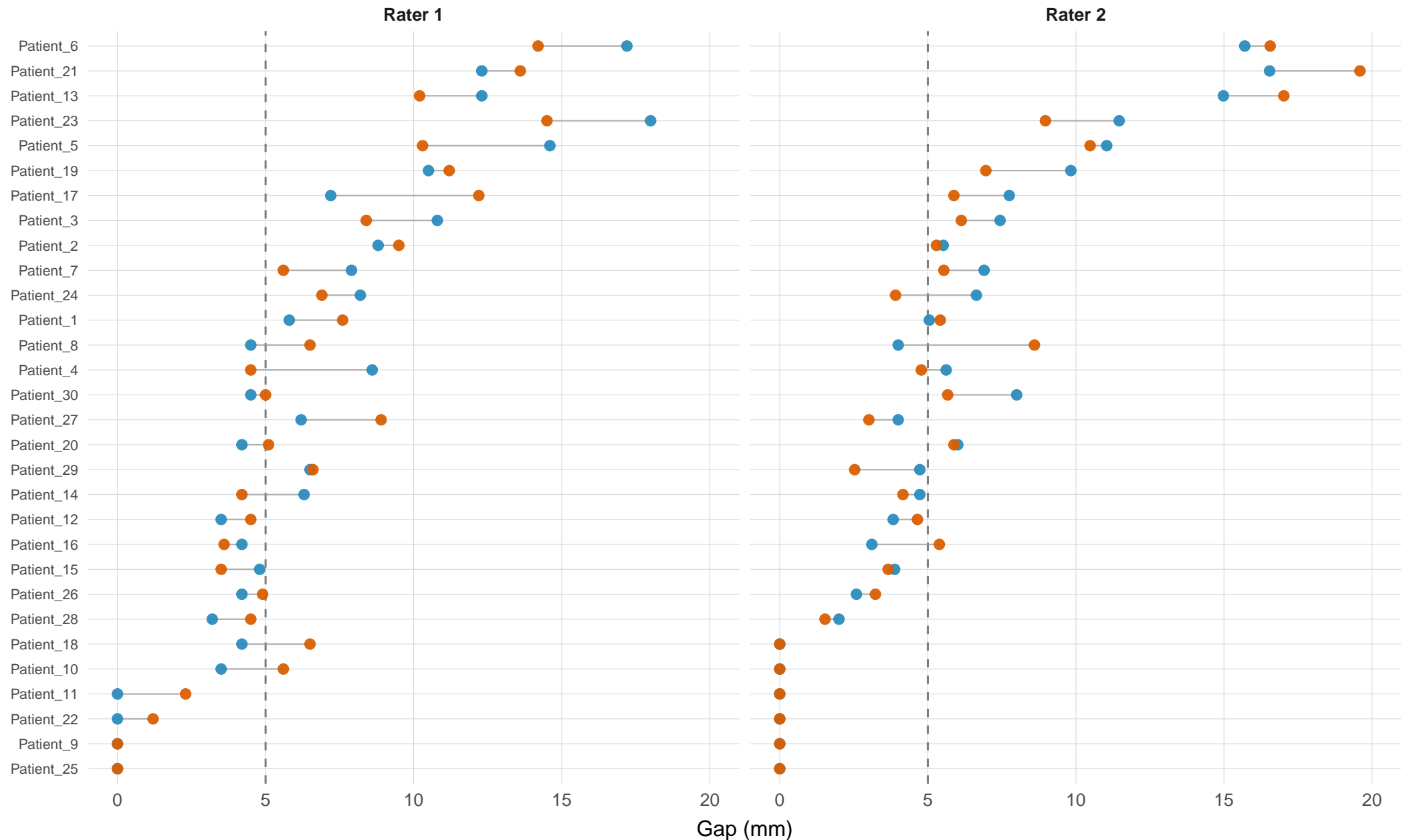

Supplement: Supplementary file 1 — Supplementary file1 (PDF 13 KB) [file 256_2026_5243_MOESM1_ESM.pdf]
