# Supplementary material for: Ultrasound gap measurement after acute Achilles rupture is reliable overall but uncertain near a 5—mm decision threshold
Source: Skeletal Radiol. 2026 May 8;55(9):2301–13. doi: 10.1007/s00256-026-05243-x (PMC13369784; doi:10.1007/s00256-026-05243-x)
Supplement: Supplementary file 2 — Supplementary file2 (PDF 21 KB) [file 256_2026_5243_MOESM2_ESM.pdf]

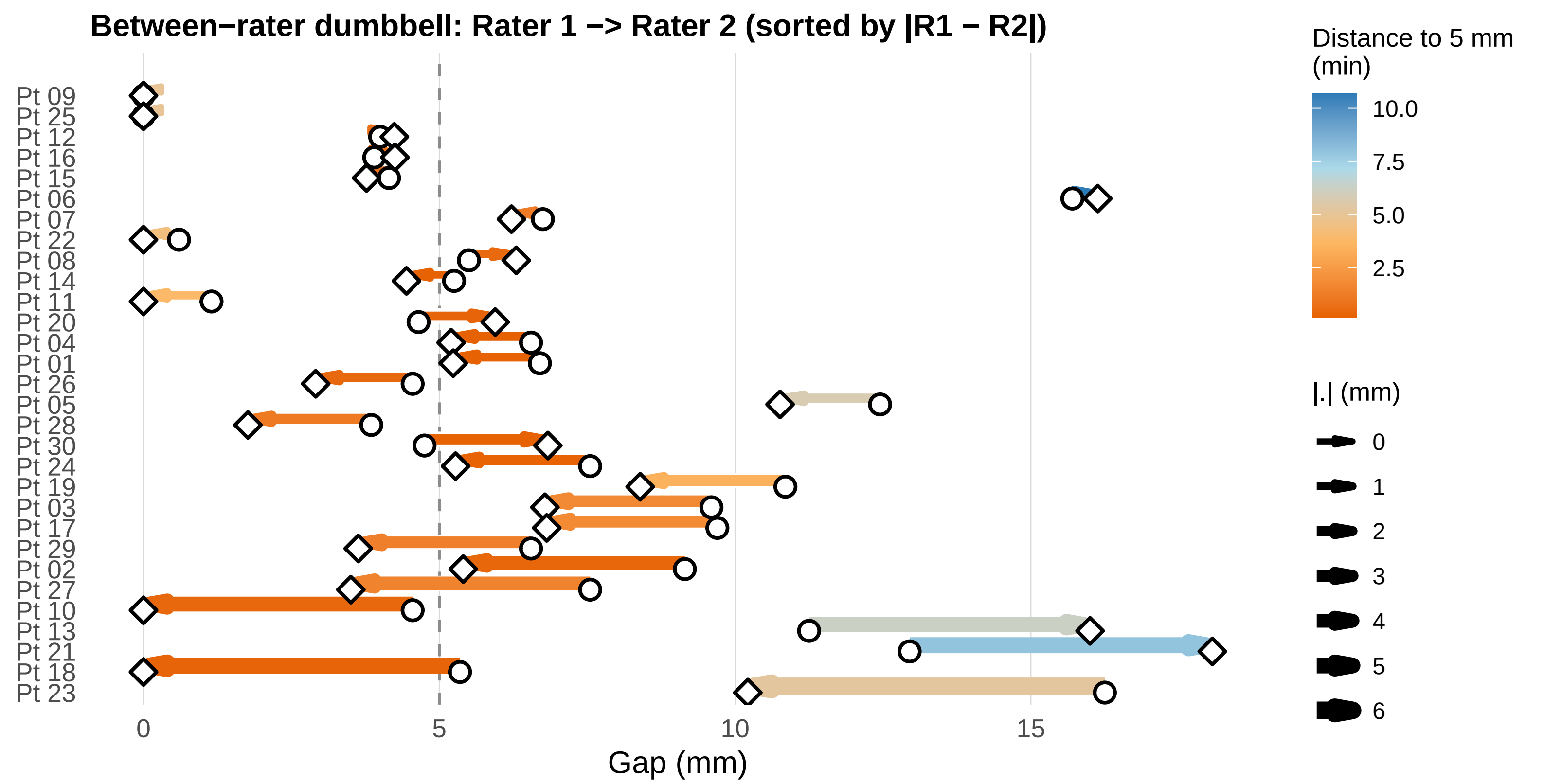
Between−rater dumbbell: Rater 1 −> Rater 2 (sorted by |R1 − R2|)
Pt 09
Pt 25
Pt 12
Pt 16
Pt 15
Pt 06
Pt 07
Pt 22
Pt 08
Pt 14
Pt 11
Pt 20
Pt 04
Pt 01
Pt 26
Pt 05
Pt 28
Pt 30
Pt 24
Pt 19
Pt 03
Pt 17
Pt 29
Pt 02
Pt 27
Pt 10
Pt 13
Pt 21
Pt 18
Pt 23
0
5
10
15
Gap (mm)
Distance to 5 mm (min)
10.0
7.5
5.0
2.5
|.| (mm)
0
1
2
3
4
5
6

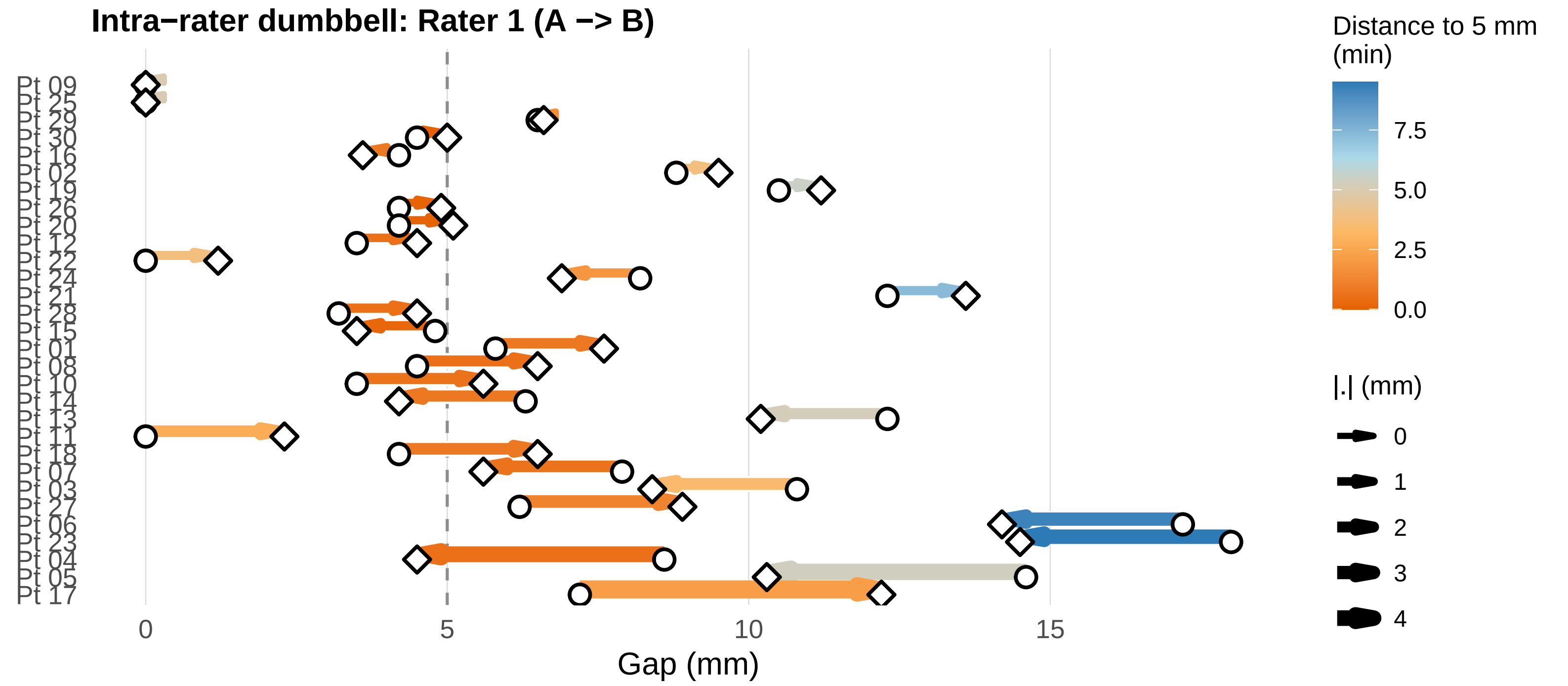
Intra−rater dumbbell: Rater 1 (A −> B)
Pt 09
Pt 25
Pt 29
Pt 30
Pt 16
Pt 02
Pt 19
Pt 26
Pt 20
Pt 12
Pt 22
Pt 24
Pt 21
Pt 28
Pt 15
Pt 01
Pt 08
Pt 10
Pt 14
Pt 13
Pt 11
Pt 18
Pt 07
Pt 03
Pt 27
Pt 06
Pt 23
Pt 04
Pt 05
Pt 17
0
5
10
15
Gap (mm)
Distance to 5 mm (min)
7.5
5.0
2.5
0.0
|.| (mm)
0
1
2
3
4

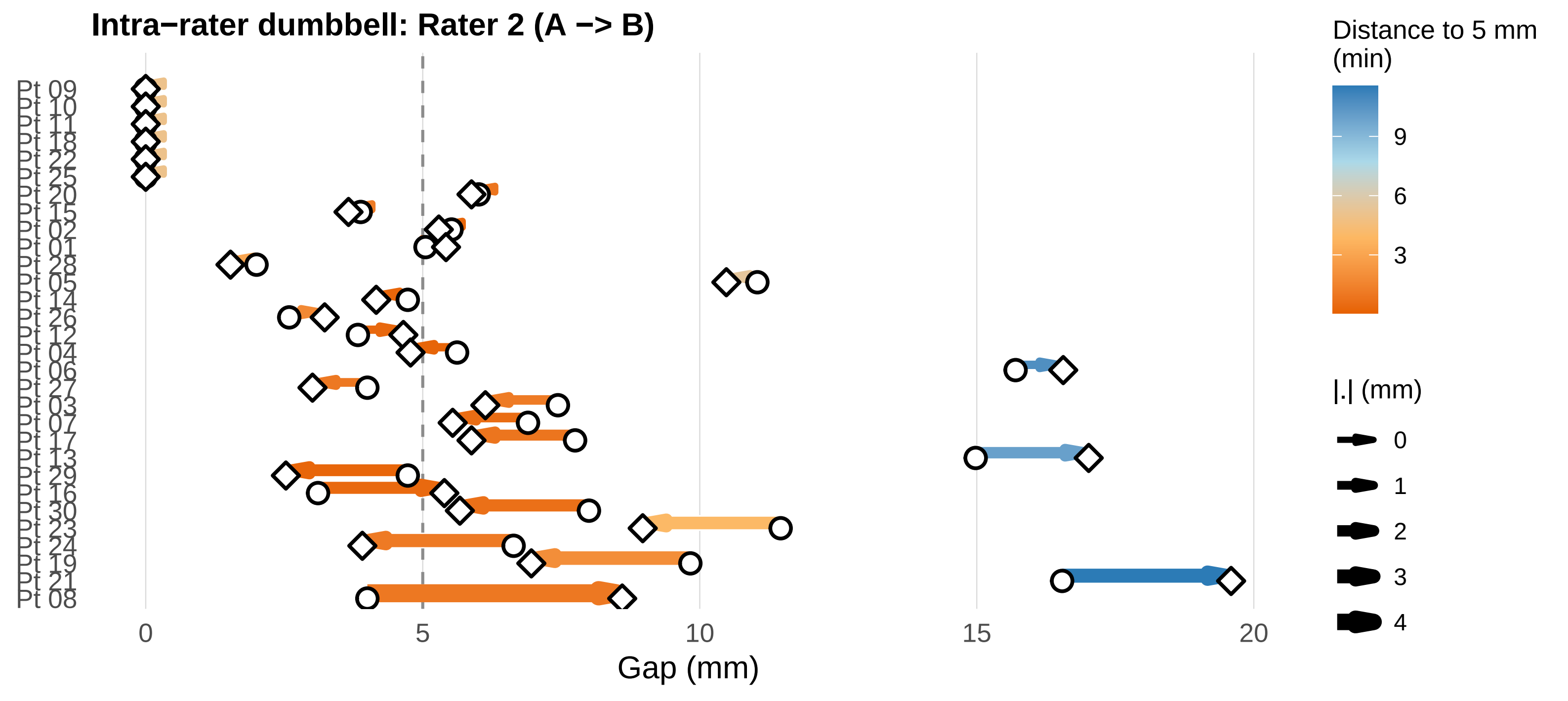
Intra−rater dumbbell: Rater 2 (A −> B)
Pt 09
Pt 10
Pt 11
Pt 18
Pt 22
Pt 25
Pt 20
Pt 15
Pt 02
Pt 01
Pt 28
Pt 05
Pt 14
Pt 26
Pt 12
Pt 04
Pt 06
Pt 27
Pt 03
Pt 07
Pt 17
Pt 13
Pt 29
Pt 16
Pt 30
Pt 23
Pt 24
Pt 19
Pt 21
Pt 08
0
5
10
15
20
Gap (mm)
Distance to 5 mm (min)
9
6
3
|.| (mm)
0
1
2
3
4
